# Photosynthetic Biogas Upgrading Using Microalgal–Bacterial Consortia: Fundamentals, Process Optimization and Challenges

**DOI:** 10.3390/microorganisms14040735

**Published:** 2026-03-26

**Authors:** María del Rosario Rodero, Loreta Drazdienė, Raúl Muñoz

**Affiliations:** 1 Institute of Sustainable Processes, University of Valladolid, 47011 Valladolid, Spain; mariarosario.rodero@uva.es; 2 Department of Chemical Engineering and Environmental Technology, University of Valladolid, Dr. Mergelina s/n, 47011 Valladolid, Spain; 3 Department of Environmental Protection and Water Engineering, Vilnius Gediminas Technical University, LT-10223 Vilnius, Lithuania; loreta.drazdiene@vilniustech.lt

**Keywords:** bioenergy, biogas upgrading, CO_2_ removal, microalgae biostimulation, photobioreactor

## Abstract

Biogas is a key renewable energy vector that can support the transition toward a net-zero carbon economy. Its direct use as a natural gas substitute is limited because it must be upgraded to meet CH_4_ purity specifications required for injection into the gas grid or for use as a vehicle fuel. This review summarizes current progress in photosynthetic biogas upgrading, an emerging biotechnology based on the symbiotic action of microalgal–bacterial consortia capable of supporting gas purification with nutrient recovery in a single integrated process. This biotechnology relies on two stages: an absorption unit that enables gas–liquid mass transfer of the biogas pollutants, and a photobioreactor in which CO_2_ and other contaminants are removed. Optimal system performance is strongly influenced by the liquid to gas (L/G) ratio, with values between 0.5 and 1.0, typically balancing effective CO_2_ removal and limited CH_4_ dilution. High-alkalinity nutrient media (1.5–2.5 gIC L^−1^) and pH > 9 remain essential to sustain the chemical gradients driving CO_2_ mass transfer. Robust microalgae/cyanobacteria such as *Chlorella vulgaris* and *Pseudanabaena* sp. frequently dominate these systems. Recent efforts in the biostimulation of photosynthesis are presented based on their potential to enhance biomass productivity and CO_2_ removal, which could decrease the footprint of the process and facilitate its large-scale adoption for biomethane production.

## 1. Introduction

The transition toward a low-carbon energy system is driven by the urgent need to address climate change, reduce the current dependence on fossil fuel-based sources and decrease negative environmental impacts. In this context, the conversion of waste into energy provides a dual advantage by generating renewable energy vectors while decreasing the environmental impacts associated with waste disposal contributing to circular economy [1]. The integration of waste-to-bioenergy technologies is essential for achieving the European Green Deal goal of a net-zero carbon economy by 2050 and for preventing global temperatures from rising above 1.5 °C. Among these technologies, anaerobic digestion represents a key biological platform for the cost-effective treatment of residues with high organic loads, with biogas generation as a primary outcome [2,3].

Biogas can be potentially applied as a renewable energy carrier due to its high methane content (50–70% v v^−1^). However, biogas yield and composition are governed by the intrinsic properties of the organic waste, including composition and redox state, and operational parameters of anaerobic digesters such as pH, organic loading rate and hydraulic retention time, which have a direct effect on the microbial performance [4]. In addition to methane, raw biogas contains carbon dioxide as the second major compound (30–50% v v^−1^), along with a range of minor compounds, such as water (<10% v v^−1^), nitrogen (<3% v v^−1^), oxygen (<1% v v^−1^), hydrogen sulfide (<10,000 ppm), ammonia (<100 mg m^−3^), volatile methyl siloxanes (<40 mg m^−3^), and volatile organic compounds (VOCs) [5,6]. The applications of biogas vary according to regional regulatory conditions, economic drivers, and the level of gas network development [7]. Biogas has been widely utilized for producing electricity and heat via cogeneration systems in diverse industrial processes. In recent years, biogas has gained increasing attention as a renewable fuel for the transport sector. For this application, it must undergo upgrading (CO_2_ removal) and purification processes that remove the other biogas components, producing biomethane with a CH_4_ content of at least 90% v v^−1^. This biomethane exhibits chemical properties nearly identical to conventional natural gas, enabling its seamless integration into existing gas networks or as a vehicle fuel in accordance with the European standards EN 16723-1:2017 and EN 16723-2:2017 [8,9]. Within the EU’s energy and climate strategy, biomethane has emerged as a strategic resource to strengthen energy independence, support the 2030 emissions reduction goals, and contribute to the long-term objective of climate neutrality by 2050.

A wide variety of technologies aimed at biogas upgrading are already available on a commercial scale. Most of them are robust and well-known physicochemical processes that are also applied in other industrial contexts. Scrubbing or the transfer of the CO_2_ into a liquid phase, has been one of the most widely used technologies, since it exhibits high efficiency, is easy to implement, and is well established [10]. Different absorbents can be employed, such as water, alkaline chemicals (e.g., alkanol amines, hydroxides, carbonates), and organic solvents (e.g., methanol or dimethyl ethers of polyethylene glycol). Depending on the type of absorbent and their CO_2_ affinity, different operating conditions (e.g., pressure) are required in the scrubbers to achieve effective separation. CO_2_ can also be removed from biogas by selectively adhering to a solid adsorbent (e.g., zeolite, activated carbon or alumina, or polymeric sorbents) in a process called pressure swing adsorption [11]. Cryogenic separation, based on the differences in the liquefaction temperatures of biogas constituents, is a technology still under development that entails high energy consumption [12]. Otherwise, the application of membranes, that retain CH_4_ while CO_2_ passes through the barrier, has grown exponentially over the past years, representing nearly 53% of the new biogas upgrading plants implemented in Europe in 2023 [7]. Despite the advantages of these physicochemical processes such as high efficiency, maturity and compact design, they still involve significant energy and/or chemical consumption [13]. Moreover, while they separate CH_4_ from CO_2_, they do not convert this CO_2_ into valuable products, which must still be managed afterwards.

Biological technologies offer not only a lower impact alternative but also a CO_2_ transformation into added value compounds or energy. In biological methanation, additional H_2_ is added to convert the CO_2_ from biogas into CH_4_ by hydrogenotrophic methanogens [14]. The sustainability of this process relies on the generation of H_2_ via water electrolysis powered by a surplus of renewable energy [15]. However, this green energy currently is not consistently available to guarantee a stable supply of H_2_ for biological methanation [16]. On the other hand, photosynthetic biogas upgrading has emerged as a highly promising solution, as it enables both the transformation of biogas into biomethane and the simultaneous capture and utilization of CO_2_ and the nutrients from digestate by microalgae/cyanobacteria. This biotechnology provides a sustainable and biological approach to valorize biogas, while simultaneously producing valuable biomass as a co-product.

The present study aims to address the fundamental principles governing photosynthetic biogas upgrading and to describe the current state of the art of this technology. In addition, it remarks main operational and environmental aspects and evaluates how different nutrient sources affect system efficiency, in order to discern the most suitable conditions for photosynthetic biogas upgrading. The main microalgae and bacteria population present in these systems are also discussed, to clarify the biological interactions in the photobioreactors and to identify which microalgal species are most suitable for photosynthetic biogas upgrading. Emerging strategies to boost CO_2_ capture and removal, such as the use of phytohormones or nanoparticle (NP) supplementation, are equally revised. Recent progress in scaling up photosynthetic upgrading technology and projects are subsequently analyzed. Finally, an assessment of the economic, technological, and environmental prospects and challenges that will shape the future implementation of this biotechnology is presented.

## 2. Principles of Photosynthetic Biogas Upgrading

**Figure 1:**
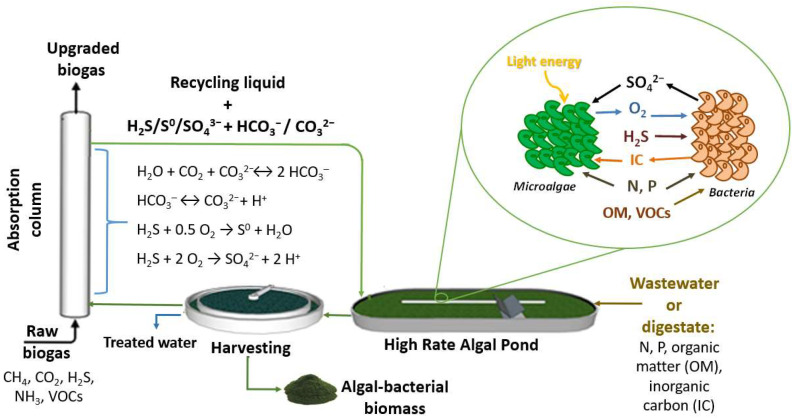
Photosynthetic biogas upgrading in an open high rate algal pond interconnected to a biogas scrubbing column.

The operation of photosynthetic biogas upgrading is governed by an interconnected set of processes in which physical gas–liquid transfer, chemical equilibrium among carbonated species, and microbial metabolism act simultaneously to determine overall system performance. The biological component relies on the synergistic interaction between aerobic bacteria and microalgae in photobioreactors, with high rate algal ponds (HRAPs) being the most widely used photobioreactor configuration based on their simplicity and low energy demand (Figure 1). Bacteria can perform the aerobic oxidation of reduced compounds from biogas (e.g., H_2_S, NH_3_, VOCs), whereas microalgae consume CO_2_, present in the cultivation broth as different dissolved inorganic carbon (IC) species, supporting bacterial activity through oxygen production via oxygenic photosynthesis. In this process, CO_2_ fixation is catalyzed by RuBisCO enzyme and supported by electrons generated during the light-driven photolysis of water. In fact, the typically high pH of the cultivation broth, which promotes the previous CO_2_ absorption from biogas, favors the conversion of CO_2_ into bicarbonate and carbonate, the primary IC forms assimilated by microalgae [17]. Through the activity of carbonic anhydrase under low CO_2_ concentration (<3%), bicarbonate is shifted back into CO_2_ for fixation by RuBisCO, while the resulting hydroxide ions react to regenerating carbonate, thereby influencing the IC equilibrium within the cultivation broth [18]. This biologically driven restoration of carbonate helps maintain the gradient required for continuous gas–liquid CO_2_ transfer. Biological IC fixation kinetics depend strongly on environmental factors such as light, temperature and nutrient availability among others, which determine the rate at which microalgae can assimilate IC. For this reason, the CO_2_ transferred into the culture must be balanced with the reactor actual CO_2_ fixation capacity, given that synthesizing 1 g of microalgal biomass requires approximately 1.83 g of CO_2_ [19]. Otherwise, IC may accumulate in the medium (thus decrease the pH and ultimately the gas–liquid mass transfer) or be released to the atmosphere, diminishing the environmental benefits of this biotechnology.

Furthermore, H_2_S can be transformed into either elemental sulfur (S^0^) or sulfate (SO_4_^2–^) through chemical pathways or via biological activity mediated by sulfur-oxidizing bacteria, which also require IC as a carbon source for growth. High photosynthetic performance promotes the full conversion of H_2_S into SO_4_^2−^, because of high oxygen availability in the broth. Neither S^0^ nor SO_4_^2−^ exerts detrimental effects on the microalgal–bacterial consortium. Indeed, sulfate can be assimilated by microorganisms as a sulfur source. Nevertheless, H_2_S concentrations above 100 parts per million by volume (ppm_v_) may negatively affect microalgal development, depending on the microalgal strain. Although biogas streams usually contain higher H_2_S levels, its rapid oxidation, together with the dilution caused by the high liquid volume in the photobioreactor, prevents inhibitory effects on microalgal metabolism. Furthermore, VOCs in biogas can be removed through oxidative processes carried out by heterotrophic bacteria, while ammonia is oxidized to nitrite by ammonia-oxidizing bacteria and subsequently to nitrate by nitrite-oxidizing bacteria within algal–bacterial photobioreactors [20].

Besides using compounds from biogas, microalgae and bacteria also need water and additional nutrients for growth. The use of synthetic mineral medium is not feasible due to the large amount (and associated cost) of water and nutrients required. An alternative is the integration of the treatment of raw wastewater or anaerobic effluents with the upgrading process [21]. In this approach, these effluents generally do not require specific pretreatment for their application in biogas upgrading, as additional processing would increase operational costs. However, basic conditioning steps, such as the removal of large solids or the separation of the suspended solid fraction in the case of anaerobic effluents, are still necessary to ensure proper system operation. Although the use of raw wastewater or liquid anaerobic effluents may introduce a diverse microbial community, including anaerobic bacteria, their impact is negligible, as they are unable to proliferate under the illuminated, high pH and oxygen-rich conditions of photobioreactors used for biogas upgrading. Overall, these streams supply the essential resources for growth and improve the overall economic and environmental footprint of the cultivation process.

The application of this produced algal–bacterial biomass, with high nutrient content and bioactive compounds, may also contribute to enhance the overall process economics. However, a key limitation for its wider application arises from the use of wastewater-derived effluents that may pose potential health risks. Consequently, this biomass is unsuitable for high-value markets such as the cosmetic, pharmaceutical, or food industries. Nevertheless, alternative uses such as bioenergy generation (e.g., biogas production) or application as a biofertilizer or biostimulant remain viable options [22]. In the latter case, pathogens, heavy metals and emerging contaminants must be assessed in accordance with applicable regulations to ensure agricultural safety.

Apart from the photobioreactor used for pollutants biodegradation, an additional gas–liquid contacting unit, typically a bubble column, is required to promote the absorption of biogas contaminants into the liquid phase [23]. The lower aqueous solubility of CH_4_ (Henry’s law coefficient (C_L_/C_G_) ≈ 0.03 at 25 °C) compared to all other major biogas components, facilitates the selective absorption of the other major biogas constituents, while CH_4_ remains predominantly in the gas phase [24]. In this regard, H_2_S is typically removed almost completely due to its relatively high aqueous solubility (C_L_/C_G_ ≈ 2.44 at 25 °C) and low inlet concentration. Notably, the oxidation of H_2_S is frequently observed in this unit. Other biogas trace contaminants, such as NH_3_, also exhibit high solubility in water and are efficiently transferred to the liquid phase. However, CO_2_ remains the main limitation of this step, as its comparatively low solubility in water (C_L_/C_G_ ≈ 0.83 at 25 °C) significantly constrains absorption-based removal. In this context, the absorption column design, together with the biogas dispersion system and operating conditions govern the volumetric gas–liquid mass transfer coefficients (k_L_a) of biogas contaminants, and thus their removal efficiency. Moreover, elevated IC concentration in the cultivation broth helps to maintain the alkaline pH (>9) necessary to sustain high the thermodynamic gradient driving CO_2_ absorption. Under such alkaline conditions, the distribution of carbonate species shifts according to their pKa values (Equations (1) and (2)), increasing the proportion of bicarbonate and carbonate ions. This shift reinforces the system’s buffering capacity and promotes the dissolution of CO_2_, whose acidic nature requires a highly alkaline medium to sustain efficient gas–liquid transfer along the absorption column. Indeed, a recent study indicate that, under near-atmospheric conditions and at pH of 9, CO_2_ shows a 2.5–3-fold higher apparent solubility in alkaline cultivation media than in water due to its conversion into bicarbonate and carbonate species [25]. This gain is partly counteracted by the salting-out effect, which reduces the physical solubility of CO_2_ in high-alkalinity solutions [26]. In contrast, methane becomes roughly half as soluble as in water because of the medium’s ionic strength. These findings support the use of alkaline media to selectively separate CO_2_ and upgrade biogas to biomethane.

(1) H_2_CO_3_ ↔ HCO_3_^−^ + H^+^  pKa_1_ = 6.35 (25 °C)

(2) HCO_3_^−^ ↔ CO_3_^2−^ + H^+^  pKa_2_ = 10.33 (25 °C)

## 3. Key Design and Operational Parameters

The efficiency and sustainability of microalgae-based biogas upgrading are influenced by multiple factors, which may impact the quality of the biomethane, nutrient recovery and pollutant removal in the cultivation broth, highlighting the importance of their understanding and optimization.

### 3.1. Main Operational and Environmental Parameters

**Figure 2:**
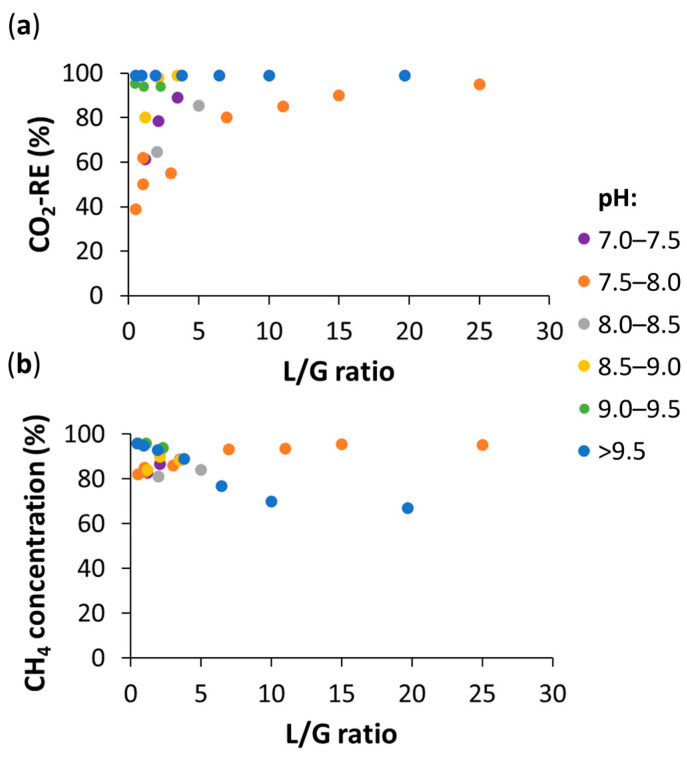
Effect of the L/G ratio under different pH conditions on biogas upgrading performance: (**a**) CO_2_ removal efficiency (RE), and (**b**) CH_4_ concentration in the upgraded biogas.

In photosynthetic biogas upgrading, operational and environmental parameters critically influence system performance. Among them, the ratio liquid flowrate/biogas flowrate (L/G) in the absorption column plays a key role on the final quality of the upgraded biomethane. High L/G ratio enhances the absorption efficiency of CO_2_ and H_2_S by preventing the acidification of the scrubbing broth as it flows through the absorption column, thereby maintaining a high concentration gradient for these acidic gases. Nevertheless, a high L/G ratio simultaneously promotes the stripping of dissolved O_2_ and N_2_ from the scrubbing broth into the biogas stream, which dilutes the CH_4_ content of biomethane and often prevents the upgraded gas from meeting the international standards for gas grid injection or vehicle fuel. In this regard, Rodero et al. [21] demonstrated in a demo system with a 150 L bubble column that while an L/G of 3.5 achieved a CO_2_ removal efficiency as high as 99.1%, the CH_4_ concentration in the upgraded biogas was limited to 88.2% due to a 11.4% concentration of stripped N_2_ and O_2._ In this experiment, the concentration of CH_4_ obtained was higher (90.3%) at an L/G of 2.1 with a CO_2_ removal of 97.8%. In addition, Toledo-Cervantes et al. [27] demonstrated that extremely high L/G ratios are counterproductive: increasing the ratio from 1 to 20 in an indoor HRAP caused the CH_4_ concentration to drop from 95% to 68%. Bose et al. [28] identified an optimal L/G ratio between 0.6 and 0.7 to ensure CO_2_ removal above 97% while keeping O_2_ levels below 1%. It is also important to note that the feasibility of operating at these lower L/G ratios is highly dependent on pH of the cultivation broth and the alkalinity required to maintain it along the absorption column. For instance, optimal L/G ratios between 0.8 and 1.3 were identified at a cultivation broth pH of 9.50 and an IC concentration of ~1900 mg L^−1^ in a 150 L bubble column, while under the same alkalinity conditions, when the pH was decreased to 9.05, these optimal L/G ratios increased to 2.1–2.4 [29]. Usually, at pH values below 8.5, high L/G ratios (>5) are required to enhance CO_2_ removal efficiency (Figure 2a). In contrast, at higher pH values, L/G ratios are lower, since pH already promotes CO_2_ mass transfer, while increasing the liquid flow rate lead O_2_ and N_2_ stripping, thereby reducing the CH_4_ concentration in the upgraded biogas (Figure 2b).

Gas residence time (GRT) or gas flowrate in the absorption column is another key operational parameter to consider. Higher GRT values enhance CO_2_ gas–liquid mass transfer, yet they require larger bubble columns and/or a greater number of columns, thus increasing capital and operating costs. GRTs higher than 30 min are commonly reported (Table 1). However, Rodero et al. [21] reduced the GRT from 30 to 20 min and observed no effect on biomethane quality. Recently, a complete H_2_S removal has been reported by Rocher-Rivas and co-workers at minimum GRT of 10 min at a pH~8 [30]. A low CO_2_ removal efficiency was observed in this system due to its inherently slower absorption kinetics and lower solubility compared to H_2_S (Table 1). As a consequence, CO_2_ requires substantially longer gas–liquid contact times to achieve effective mass transfer and subsequent removal from the biogas stream. Nevertheless, reducing GRT to values below 6 min remains necessary to enable photosynthetic biogas upgrading feasible and competitive on an industrial scale [28].

Environmental conditions are also key factors governing the performance of photosynthetic biogas upgrading, particularly for the biological stage of the process. Light availability governs the efficiency of photosynthetic biogas upgrading, as it provides the energy required for CO_2_ fixation into microalgal biomass. Increasing light intensity enhances CO_2_ fixation up to the saturation point, beyond which photoinhibition may occur, reducing biomass productivity [37]. For instance, light intensities of 162 µmol m^−2^ s^−1^ produced a 5-fold increase in biomass compared to 20 µmol m^−2^ s^−1^ under the same photoperiod [38]. Both light intensity and photoperiod significantly influence process performance, since dark periods promote endogenous biomass consumption and may lead to CO_2_ stripping, limiting environmental benefits [39]. A 75% reduction in biomass concentration and CO_2_ fixation capacity was observed when the duration of the light period was shortened from 24 h to 14 h in *Aphanothece microscopica* Nägeli cultures exposed to a light intensity of 150 μmol m^−2^ s^−1^ [40]. Otherwise, a 14:10 h light/dark photoperiod was optimal for *C. vulgaris* at a moderate light intensity of 450 μmol m^−2^ s^−1^, showing superior CO_2_ removal efficiency and nutrient uptake from digestate compared with both 12:12 and 16:8 light/dark photoperiods [41]. In this context, while short photoperiods result in progressively reduced growth rates and CO_2_ fixation, the optimal light–dark cycle ultimately depends on both the light intensity and the specific microalgal strain. Additionally, light conditions affect the synthesis of value-added products, and optimal wavelengths may vary depending on the microalgal species. The choice of light source strongly impacts system efficiency and economics. Although LED lights offer advantages such as narrow spectral output, low heat generation and high energy efficiency, their capital and operational costs can be prohibitive due to the high surface of photobioreactors, substantially increasing total energy consumption [42,43]. Consequently, sunlight remains the only cost-effective and sustainable option for large-scale photosynthetic biogas upgrading. Nevertheless, its variability due to season and day–night cycles require adaptive strategies. In this regard, hybrid systems combining sunlight with LED supplementation and renewable energy sources (e.g., solar panels and wind turbines) have been proposed to ensure stable and continuous illumination [44]. Nonetheless, while these systems may be suitable for high-value microalgal production in closed photobioreactors, they are not appropriate for HRAP aiming to biogas upgrading, where capital costs would increase substantially and the sales of biomethane might not compensate the initial investment. On the other hand, temperature affects both microalgal–bacterial activity rates and the solubility of biogas contaminants. For most microalgae species, the optimal growth temperature typically ranges from 15 °C to 30 °C, though the precise optimum is inherently strain-dependent [45,46]. As temperature rises within this interval, metabolic activity tends to intensify, leading to faster CO_2_ assimilation by microalgae supporting more efficient oxidation of H_2_S. However, when temperatures surpass the limits tolerated by microalgae species, biomass productivity declines sharply, which is a common issue in outdoor systems exposed to seasonal or daily fluctuations in irradiation. Apart from the biological impact, temperature is inversely correlated to the aqueous solubility of gases. Nevertheless, while temperature variations significantly impact microalgal productivity, its effect on CO_2_ removal efficiency is generally minimized in systems utilizing high-alkalinity broths (IC > 1500 mg L^−1^), which provide a robust buffer against these environmental fluctuations.

### 3.2. Composition of the Nutrient Source

The selection of an appropriate cultivation medium, particularly using residual water sources, is fundamental for the cost-efficiency and environmental sustainability of photosynthetic biogas upgrading. Residual effluents not only provide a low-cost source of nutrients but also strongly influence the overall absorption performance, influencing both the chemical equilibrium of the scrubbing liquid and the overall effectiveness of contaminant removal (Table 1). High-alkalinity effluents, such as the liquid fraction of the digestates (centrates in the context of wastewater treatment plants), are particularly advantageous for biogas upgrading as they contain significant concentrations of bicarbonates and carbonates (500–2000 mgC L^−1^). In addition, the high ionic strength of centrates provides a secondary hydrodynamic advantage by preventing bubble coalescence, increasing the gas–liquid interfacial area and improving the overall mass transfer coefficient (K_L_a) within the absorption column [47].

In this context, the substitution of domestic wastewater, which typically has a low alkalinity, with nutrient-rich centrate, allowed CO_2_ removal efficiencies to increase from approximately 90% to a peak of 99%, significantly enhancing the final biomethane quality [21]. However, to ensure consistent CO_2_ absorption and prevent the rapid acidification of the recirculating scrubbing broth along the absorption column, without requiring excessive liquid flow rates, the most optimal cultivation conditions generally involve IC concentrations between 1.5 and 2.5 g L^−1^ [48]. For this reason, carbonate solutions are often added to medium-strength digestates (500–1000 mg IC L^−1^) to increase their buffer capacity. Furthermore, although high-strength agroindustrial streams, such as piggery wastewater, provide higher alkalinity compared to centrates, they also contain elevated concentrations of organic matter, ammoniacal nitrogen and other toxic compounds such as heavy metals that can inhibit microalgal performance and lead to excessive turbidity [49]. High turbidity and dark coloration limit light penetration into the culture medium, which in turn decreases the photosynthetic activity of microalgae. In such cases, dilution or pretreatment to remove color or toxic components from these effluents is often necessary to prevent adverse effects on microalgae growth.

The use of digestates and/or high-alkalinity media also presents other technical challenges. The high pH levels required for biogas upgrading can foster chemical precipitation of essential nutrients like phosphorus and magnesium, potentially slowing microalgal metabolic rates if the residual source is not properly balanced. Additionally, elevated ammonium levels in certain digestates (up to 3.0 g L^−1^) can be toxic to microalgae at the high pH prevailing in these high-strength wastewaters [50]. In order to prevent microbial inhibition, low hydraulic loading rates are applied when operating with these effluents, which often promotes substantial evaporative losses in HRAPs. Under these conditions, the system can effectively function without generating a liquid discharge, and in some cases, additional make-up water must be supplied to offset the water volume lost through evaporation. Nevertheless, integrating biogas upgrading with the bioremediation of residual streams facilitates a circular bioeconomy model, significantly reducing the energy and chemical penalties, and the CO_2_ footprint, associated with traditional physical–chemical upgrading technologies.

### 3.3. Microalgal–Bacterial Population

Photobioreactors, in particular HRAPs, devoted to the integration of digestate treatment with biogas upgrading, host microalgal species alongside a highly diverse prokaryotic community, both functioning in a tightly coordinated symbiotic relationship. The efficiency of photosynthetic biogas upgrading depends heavily on selecting microalgae capable of thriving under the stress of the system, such as alkaline pH (9–11), temperature changes, high concentrations of IC and the presence of H_2_S or other toxic compounds. Although HRAPs may be inoculated with mixed cultures, some specific species such as *Chlorella vulgaris*, *Mychonastes homosphaera* and *Chloroidium ellipsoideum* often emerge as the dominant species due to their extreme versatility and resilience to wastewater pollutants (Table 2). Other effective species include *Scenedesmus obliquus*, which frequently demonstrates higher CO_2_ fixation rates and cell densities in wastewater environments than *C. vulgaris* [51,52]. These microorganisms are particularly suited for this role because they utilize carbon concentrating mechanisms and the enzyme carbonic anhydrase to efficiently assimilate bicarbonates at high pH levels while regenerating carbonates for continuous CO_2_ absorption [53].

In low temperature climates or high-alkalinity environments, filamentous cyanobacteria like *Anabaena* sp. and *Phormidium* sp. have been considered the best candidates. These species are not only able to grow under these harsh conditions, but they also offer a significant operational advantage because their morphology enables easier and more cost-effective harvesting compared to unicellular algae. Furthermore, a recent work has highlighted an alkaliphilic strain of *Desmodesmus subspicatus* as a viable option for photosynthetic biogas upgrading. This microalga was shown to maintain steady growth under pH between 9 and 10 and elevated IC levels (1.5–2.5 g L^−1^) at 20 °C, underscoring its potential for use in such systems [54]. Additionally, alkalihalophilic species such as *Arthrospira (Spirulina) platensis* are valued for their ability to thrive in high-carbonate environments while producing valuable co-products like protein, pigments and fatty acids [55,56]. Seasonal variations also drive population shifts. For example, while green algae dominate during high light periods, cyanobacteria such as *Leptolyngbya* sp. and *Pseudanabaena* sp. can outcompete them under lower light intensities [57].

**Table 2:** Microalgal–bacterial community composition under different conditions during photosynthetic biogas upgrading.

Experimental Set-Up	Inoculum	Biogas Composition (% v v^−1^)	Nutrient Source	pH	Microalgal/Cyanobacterial Species	Bacterial/Other Community	Biomass Productivity (g m^−2^ d^−1^)	Ref
Outdoor HRAP (180 L) + bubble column (2.5 L)	Microalgal consortium: *Leptolyngbya lagerheimii* (54%), C. vulgaris (28%) and other + activated sludge	CO_2_ (29.5), H_2_S (0.5) and CH_4_ (70)	Centrate	9.0–9.8	*Chlorella vulgaris* (dominant in almost all stages), *Chlorella kessleri*, *Westella* sp, *Tetradesmus obliquus*, *Leptolyngbya lagerheimii*, *Pseudanabaena* sp. *	Chemo-organoheterotrophs (i.e., *Pseudomonas, Trichococcus*), anoxygenic photosynthetic bacteria (*Chromatiaceae* (family) and *Rhodobacter*), denitrifiers (*Aquimonas, Acinetobacter, Comamonas, Caldilinea, Rhodobacteraceae*), nitrifying bacteria (*Nitrospira, Nitrosomonadaceae*), methanotrophs (*Methylobacillus, Methylomonas*)	7.5–22.5 (season-dependent)	[57]
Outdoor HRAP (180 L) + bubble column (2.5 L)	*Pseudoanabaena* sp. (98%), *Chlorella vulgaris* (2%)	CH_4_ (60.0), CO_2_ (38.7), N_2_ (1.0) and O_2_ (0.3)	Food waste digestate + SWW	8.4–9.6	*Pseudoanabaena* sp. (98% dominant)	N.A.	15.0–22.5	[33]
Glass photobioreactors (16.8 L)	*Chlorella vulgaris* or *Scenedesmus obliquus* + *Ganodermalucidum* fungi + activated sludge	CH_4_ (61.2), CO_2_ (34.7)	PWW diluted	~7.0	*Chlorella vulgaris*, *Scenedesmus obliquus*	*Ganodermalucidum* fungi + Nitrifying–denitrifying activated sludge	Up to 0.1 (g L^−1^ d^−1^)	[51]
Closed Tubular-PBR (132 L) + bubble column (2.5 L)	*Chlorella vulgaris*, *C. ellipsoidea*, *Tetracoccus* sp., *Pseudanabaena* sp. (79%)	CO_2_ (29.5), H_2_S (0.5) and CH_4_ (70)	Brunner medium (digestate mimic)	9.0–9.3	*Pseudanabaena* sp. (dominant 95%)	*Tetracoccus* sp. (SOB)	9.7–9.9	[34]
Indoor HRAP (180 L) + bubble column (2.5 L)	Microalgal consortium: *Gueitlerinema* sp. (61.5%), *Staurosira* sp. (1.5%), *Stigeoclonium tenue* (37%)	CO_2_ (29.5), H_2_S (0.5) and CH_4_ (70)	MSM/Centrate	9.1 ± 0.1	*Mychonastes homosphaera*, *Limnothrix planktonica*, *Phormidium* sp. and *Stigeoclonium tenue*	Genus *Blastocatella*, *Gammaproteobacteria* class and genus *Thioalbus* (SOB)	2.2–7.5	[27]
Indoor HRAP (180 L) + bubble column (2.5 L)	Microalgae-bacteria consortium *Chlorella saccharophila* (dominant)	CH_4_ (70), CO_2_ (30)	Centrate	8.6–8.7	*Chlorella saccharophila*, *Pseudanabaena* sp., *Arthrospira* sp. (minoritary).	N.A.	~22.5	[35]
Indoor HRAP (180 L) + bubble column (2.5 L) + NPs	Microalgae-bacteria consortium *Mychonastes homosphaera* (15%), *Chlorella vulgaris* (85%)	CO_2_ (29.5), H_2_S (0.5) and CH_4_ (70)	Centrate supplemented with IC	9.1–9.4	*Chloroidium ellipsoideum* (dominant at end)	N.A.	Up to 89 (with NPs)	[36]

* Only species with an abundance greater than 10% were included. HRAP: high rate algal pond; IC: inorganic carbon; MSM: mineral salt medium; N.A.: not analyzed; NPs: nanoparticles; PWW: piggery wastewater; SOB: sulfur-oxidizing bacteria; SWW: synthetic wastewater.

The prokaryotic community working alongside these microalgae in photosynthetic biogas upgrading coupled with digestate treatment is highly diverse. The bacterial community is mainly composed of groups involved in organic matter degradation from wastewaters (e.g., *Pseudomonas* and *Trichococcus*), H_2_S oxidation to sulfate from biogas (e.g., *Tetracoccus* sp., *Thioalbus* genus, *Chromatiaceae* family), anoxygenic photosynthesis (*Chromatiaceae* family and *Rhodobacter*), nitrification (such as *Nitrospira* and *Nitrosomonadaceae*) and denitrification (such as *Aquimonas*, *Acinetobacter*, and *Comamonas*) (Table 2). Although dissolved oxygen levels are high during the day due to photosynthetic activity, the anoxic or low-oxygen zones that develop at night or in the settler not only promote nitrate-to-nitrite conversion by denitrifiers, but also create favorable conditions for the growth of fermentative bacteria and purple sulfur bacteria [57]. Finally, methanotrophic and methylotrophic genera were detected in the cultivation broth of the photobioreactors fed with centrate at very low abundances (<1%), likely reflecting their presence in the centrate. Fortunately, the conditions in the photobioreactor did not allow them to proliferate without any negative effect on final methane concentration.

## 4. Novel Strategies Boosting Photosynthetic Biogas Upgrading: Microalgae Biostimulation

Photosynthetic biogas upgrading deployment at an industrial scale is largely hindered by the restricted photosynthetic activity of microalgae and inefficient CO_2_ transfer to the culture medium. In this regard, the discovery of compounds capable of stimulating microalgal growth and enhancing both their productivity and the robustness of cultures has recently begun to attract considerable attention.

Recent progress in the field of microalgal biotechnology has remarked the potential of phytohormones and other bioactive molecules to enhance microalgal physiological performance. Despite being synthesized by organisms at very low concentrations, these compounds exert broad regulatory control over cellular functions, shaping growth dynamics, metabolic activity, and responses to environmental stressors. Within this group, strigolactones and their synthetic derivatives, mainly GR24, have gained relevance for their ability to markedly influence microalgal behavior. Treatment with GR24 has been associated with enhanced metabolic efficiency, elevated photosynthetic capacity, improved cellular robustness, thus contributing to faster growth and greater biomass yields. The addition of 10^−9^ M of GR24 increased the formation of chlorophyll a in *C. vulgaris* and its productivity [58]. This addition was most effective when *C. vulgaris* growth in a co-cultivation algal-activated sludge system, outperforming both monoculture and algal–fungal co-culture conditions [59]. Overall, the addition of 10^−9^ M of GR24 in co-cultures of *Chlorella* sp. with bacteria and/or fungi has been shown to increase biomass growth by up to 52% and to enhance CO_2_ removal efficiency up to 25% compared with the control (without addition) (Table 3).

Natural strigolactones, such as 5-Deoxystrigol (5-DS) that can be obtained from legume roots, also enhance microalgal growth while offering a lower-cost alternative to synthetic ones. In this regard, the addition of 10^−11^ M of 5-DS (optimal dose) entailed an increase in daily biomass productivity of 53.4% with *Tetradesmus obliquus* in co-culture with *G. lucidum* and endophytic bacterium S395–2 (Table 3). A similar or even better impact was observed in other strains, such as *C. vulgaris* and *N. palea*, where the addition of this phytohormone under the same conditions increased biomass productivity by ~68% in both cases [61]. Nevertheless, the use of 5-DS was less effective than GR24 for the treatment of biogas and digestate using a symbiotic system of *C. vulgaris*, endophytic bacteria and *Clonostachys* fungi. Additionally, the phytohormone cytokinin kinetin (KT) can promote growth, regulate nutrient transport and coordinate stress responses in photosynthetic systems such as plants [67]. The effect of adding KT at 10^−7^ M has been evaluated for digestate treatment and biogas upgrading using *Chlorella* grown in monoculture or in co-culture with bacteria, fungi, or both. In every case, the growth rate, biomass productivity and CO_2_ removal increased relative to the control (without KT addition). Nonetheless, the enhancement in growth was lower than that achieved with GR24 [59]. In general terms, these signaling molecules may be valuable tools for improving microalgal cultivation strategies and enhancing the resilience of cultures exposed to adverse environmental conditions.

The exceptional surface-to-volume ratio and high chemical reactivity of NPs have driven significant interest in their application across both chemical and biological fields. In microalgal cultures, various types of NPs, including metal oxides like α−Fe_2_O_3_ and SiO_2_, as well as silicon carbide (SiC) and carbon-coated zero-valent iron (CACOI), have emerged as effective algal stimulants that trigger enhanced metabolic and photosynthetic activity (Table 3). The addition of CACOI NPs at 140 mg L^−1^, synthesized via hydrothermal carbonization of olive-mill wastewater from the company CALPECH, has been shown to more than double microalgal productivity, increasing it from 22.5 to 48.2 g m^−2^ d^−1^ in a pilot HRAP treating biogas and centrate. Similarly, the use of liquid CACOI NPs at 2 mL L^−1^ in a consortium of *C. vulgaris* and *M. homosphaera* promoted an even higher biomass productivity of 89 g m^−2^ d^−1^ in a similar system [36]. Other strains such as *Arthrospira platensis* reached productivities of 1.06 g L^−1^ d^−1^ when supplemented with CACOI NPs, while SiC NPs at 150 mg L^−1^ supported a cell concentration of 3.18 g L^−1^ in *Scenedesmus* sp., representing a significant rise over control cultures [68].

Apart from the above-mentioned metabolic stimulation, NPs also have a role in optimizing CO_2_ capture mainly attributed to their high specific surface area and active sites, which can eventually improve gas–liquid mass transfer. These materials facilitate the transfer of CO_2_ molecules since they are adsorbed onto the NP surface and then released into the microalgal culture medium. Indeed, a recent research indicates that the supplementation of CACOI NPs can increase the volumetric mass transfer coefficient by 44% [64]. Such improvements are critical for biogas upgrading, where CO_2_ removal efficiencies have been observed to exceed 98%, allowing for the production of high-grade biomethane with purity levels reaching 94.9% even at lower IC concentration [36]. Furthermore, polymeric nanofibers containing iron oxide NPs, used as physical adsorbents, have achieved CO_2_ biofixation rates of up to 310.9 mg L^−1^ d^−1^ under outdoors conditions, significantly outperforming the operation without NPs [62].

NP supplementation to the algal cultivation broth might entail also certain drawbacks and risks, as their effects can shift from stimulatory to toxic depending on the dosage, the microalgal species involved, and the environmental conditions in which the treated effluent is released. At high concentrations, NPs may promote the formation of reactive oxygen species, ultimately causing oxidative stress, compromising membrane integrity, and triggering cell lysis [69]. For example, exposure to high levels of α-Fe_2_O_3_ (100 mg L^−1^) has been shown to reduce the biomass of *C. vulgaris* by as much as 83.7% [70]. In addition, concentrated NP suspensions often exhibit dark coloration, which can obstruct light penetration and consequently inhibit photosynthesis due to shading effects. As a result, the concentration and delivery format of NPs need to be adjusted to maximize beneficial effects while minimizing risks to microalgal cultures and the surrounding environment.

Despite their advantage to boost microalgae, the presence of phytohormones and NPs must be considered when reusing biomass or discharging effluents since they can pose adverse environmental effects. Although phytohormones are typically applied at low concentrations and are not inherently problematic, their accumulation beyond physiological levels may pose ecological risks, with strigolactones altering soil microbial communities and strigolactone mimics such as SL-6 showing toxic and genotoxic effects on aquatic organisms at concentrations higher than 1 µg L^−1^ [71,72]. NPs could also pose environmental risks since they tend to persist due to their small size, high reactivity and high stability, which allow them to increase their mobility and long-term bioavailability, enhancing ecological risks in terrestrial and aquatic environments [73]. Studies show that these NPs can accumulate in aquatic microorganisms (e.g., across algae, crustaceans, molluscs and fish) and contribute to cellular injury and impaired physiological functions [74,75,76]. In this context, further research on their environmental fate is essential before large-scale deployment of this novel algal stimulation strategy.

## 5. Recent Advances in Scale-Up

Early evaluations of photosynthetic biogas upgrading were conducted using laboratory-scale systems, including open ponds and closed photobioreactors, operated under both indoor and outdoor conditions. These systems allowed to identify key process parameters and provided an overview of microalgal–bacterial growth and biomethane quality [77]. Nevertheless, the reduced scale of laboratory systems limited their ability to accurately reflect biogas upgrading performance. In this regard, the sustainable nature and cost competitiveness of microalgae-based biogas upgrading have attracted increasing research interest, leading to efforts in scale-up of this process within certain European projects.

**Figure 3:**
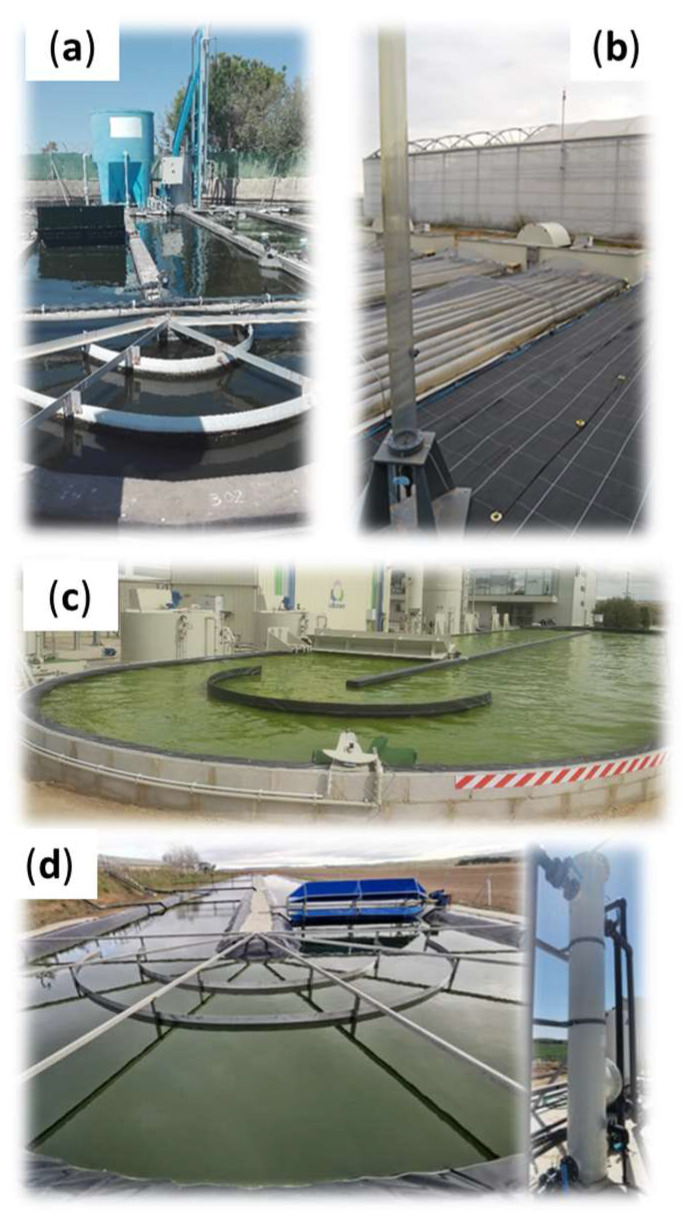
Process scale-up of photosynthetic biogas upgrading: (**a**) demo-scale HRAP within the INCOVER project; (**b**) demo-scale tubular photobioreactor within the INCOVER project; (**c**) demo-scale HRAP within the URBIOFIN project; (**d**) demo-scale HRAP within the LIFE SMARTAGROMOBILITY project.

The European project INCOVER was the first to scale-up this technology in the field of domestic wastewater treatment, using a 30 m^2^ HRAP with a working volume of 9.6 m^3^ (Figure 3a). Domestic wastewater or centrate from the anaerobic treatment of the sludge were used in this system as a nutrient and water source, obtaining a biomethane with CH_4_ concentrations reaching up to 90.6% when centrate was fed [21]. In addition, a semi-closed tubular photobioreactor was also tested within this project obtaining a biomethane with a CH_4_ content of 93.2% using agricultural wastewater supplemented with a carbonate solution as a nutrient source (Figure 3b) [78]. As a further step in the development of photosynthetic biogas upgrading, the European BBI-URBIOFIN project implemented this technology for the concomitant treatment of biogas and digestate from the municipal solid waste treatment using an 80 m^3^ HRAP with an illuminate surface of 272 m^2^ (Figure 3c). As part of the European LIFE SMARTAGROMOBILITY project, a 1008 m^2^ HRAP with an effective capacity of 320 m^3^ was deployed to treat livestock digestate and biogas, demonstrating the system’s operational performance throughout the whole year (Figure 3d) [79]. This demo system proved to be robust, even though a seasonal fluctuation in CH_4_ concentration is evident, ranging from 94% in summer to 88% in winter. These European projects, carried out at various locations throughout Spain, confirmed the cost-effectiveness of this process at a technology readiness level (TRL) of 6–7. Currently, the European project LIFE W2B will install a 50 m^3^ HRAP (160 m^2^ of surface area) interconnected with a 0.5 m^3^ biogas absorption bubble column for valorizing biogas and digestate from the co-digestion of multiple urban organic residues in Crete (Greece).

## 6. Technological, Economic and Environmental Perspectives and Challenges

Despite the huge potential of photosynthetic biogas upgrading for its integration into a biorefinery system, its application is still constrained by the limited gas–liquid mass transfer of CO_2_ from the biogas to the liquid culture medium, which often results in low-carbon capture efficiencies. Furthermore, the stripping of O_2_ and N_2_ from the scrubbing liquid to the upgraded biogas can compromise biomethane quality and, if not strictly controlled, may lead to the creation of an explosive gas mixture. To overcome these issues, the development of advanced automated control systems has allowed optimization of the L/G ratio in real time, ensuring stable biomethane concentration and improving energy efficiency associated with liquid pumping [29]. Moreover, advances in absorption column design have provided an additional boost to process efficiency [23]. In addition, innovative strategies are being deployed, such as the addition of NPs, enhancing CO_2_ removal via photosynthesis stimulation from biogas even under moderately alkaline conditions [36,80].

Geographic and climatic limitations also pose a significant challenge since microalgal and bacterial performance is highly sensitive to ambient temperature. In continental and temperate oceanic regions, low winter temperatures and reduced light availability markedly limit microalgal productivity. The use of greenhouses provides a practical solution for enabling year-round operation in cooler climates, although it increases both capital expenditure and energy demand. Moreover, selecting indigenous cold-tolerant microalgal strains or applying adaptive laboratory evolution to improve their thermal tolerance and overall performance under fluctuating environmental conditions can significantly strengthen system resilience. Furthermore, enhanced modeling tools should be applied for predicting the behavior of these complex systems under variable environmental conditions [81,82].

The extensive land requirement is perhaps the most significant hurdle for the large-scale deployment of photosynthetic biogas upgrading. HRAPs used for CO_2_ fixation demand a surface approximately 1860 times larger than conventional physicochemical units. For instance, upgrading a biogas flow of 300 Nm^3^ h requires approximately 13.4 hectares of illuminated area, making the technology difficult to implement in urban facilities with limited space [83]. Additionally, light availability represents another critical challenge, as photosynthesis efficiency is governed by the diurnal cycle and seasonal variability in irradiance. In winter, low light intensities become a limiting factor for CO_2_ assimilation. This effect is aggravated when treating effluents with high turbidity and dark coloration, which further restrict light penetration. While artificial illumination using LEDs can stabilize production, it is economically prohibitive for industrial-scale biogas upgrading plants [84]. Optimization strategies currently focus on improving light-to-volume ratios through better photobioreactor design and more uniform light distribution, ultimately increasing CO_2_ capture efficiency while reducing the required installation area [85,86].

From an economic perspective, the high land and equipment requirements result in a capital expenditure similar than conventional biogas upgrading technologies (without considering the biomass drying) [83]. This high initial investment can hinder its adoption in smaller facilities, such as pig farms, where relative costs increase exponentially at lower biogas flows. However, photosynthetic biogas upgrading offers significantly lower energy requirements and operational costs (0.03 € Nm^−3^) due to its mild operating conditions, which render this green technology more competitive than the 0.15–0.2 € Nm^−3^ typically required for physicochemical technologies [21]. In addition, the economic viability of photosynthetic biogas upgrading is significantly boosted when using a cascading biorefinery approach, where biomethane production is coupled with wastewater remediation and the recovery of high-value co-products such as pigments, bioenergy, biofertilizers or biostimulants from the algal–bacterial biomass [87]. In some scenarios, the sale of these co-products allows the system to become profitable within five years of operation [83].

Finally, photosynthetic biogas upgrading stands out for its superior sustainability, as it integrates carbon capture and utilization, with simultaneous nutrient recovery from digestate treatment. This dual functionality helps reduce the eutrophication potential of digestates while generating a low-carbon energy vector. While this process reduces greenhouse gas emissions by a factor of approximately 45 compared to conventional biogas upgrading technologies, environmental challenges remain related to its large area footprint, biomass harvesting and the potential accumulation of heavy metals or pathogens in biomass cultivated in digestates [83]. Despite these challenges, the environmental advantages, particularly the ability to utilize directly biogenic CO_2_ for the production of bioproducts and the recovery of nutrients in the form of an algal biomass that can be used as a feedstock for bioestimulant production, position this approach as a key pillar of the circular bioeconomy [88]. Continued progress in low-carbon and zero-emission biotechnologies will be crucial to enabling the commercial shift toward more sustainable, circular value chains from organic waste.

## 7. Conclusions

Photosynthetic biogas upgrading has gained recognition as a resilient and environmentally friendly alternative to conventional biogas cleaning technologies. Its core strength lies in the synergistic activity of microalgal–bacterial communities, which enables the simultaneous elimination of CO_2_ and H_2_S while recycling nutrients present in digestates. The review highlights that system performance is strongly dependent on several fundamental operational parameters, such as the L/G ratio, light availability and the characteristics of the influent, which regulate mass-transfer processes and microbial stability. Advances in biostimulation, including the use of phytohormones or metal-based NPs, have shown notable potential to enhance photosynthetic efficiency, biomass yield and/or CO_2_ removal rates.

Despite this progress, industrial-scale implementation still faces substantial challenges. Outdoor HRAPs are particularly sensitive to climatic variability and significant land requirements, all of which complicate stable year-round operation. These constraints impact directly the techno-economic and environmental performance of this platform technology, and the feasibility of integrating these systems into existing anaerobic digestion infrastructures. This review also drafted a roadmap to accelerate development of this promising biotechnology. In this context, a strong need to advance both photobioreactor engineering and gas–liquid mass-transfer systems, aiming to reduce land requirements while maximizing the efficiency, was identified. Advanced modeling is necessary to forecasting system dynamics under variable environmental conditions. Integrating automation and control strategies may ultimately enable stable, low-cost operation at commercial scale. Future work should also evaluate long-term stability and safety of algal stimulants, ensuring no unintended ecological impacts. Furthermore, coupling photosynthetic upgrading with other biorefinery processes could unlock new value chains, improving overall economic viability. Collectively, these advancements will help consolidate microalgal-based biogas upgrading as a scalable and competitive technology within the circular bioeconomy.

## Figures and Tables

**Table 1:** Process performance in photosynthetic biogas upgrading combined with wastewater treatment.

Reactor Type and Configuration	L/G Ratio	GRT (h)	Light Intensity (μmol m^−2^ s^−1^)	WW Type, pH and IC Concentration (mg L^−1^) in the Cultivation Broth	HRT (d)	CO_2_-RE (%)	H_2_S-RE (%)	CH_4_ Concentration (%)	Ref
9.6 m^3^ HRAP interconnected to a 150 L AC	1.2–3.5	0.33–0.55	-	Domestic WW; pH: 7.1–7.3; IC: 26–30	3.5–8	59–89	86–98	79–89	[21]
7 L HRAP interconnected to a 1 L AC/airlift	1.0–4.0	0.16–1.0	200	MSM; pH:8–9	5	7–38	100	66–75	[30]
300 L HRAP interconnected to a 3.2 L AC	0.5–1.0	0.38	-	MSM and leachate (15:1 *v*/*v*); pH: 8–9.5; IC: 800–2200	138	80	100	70–90	[31]
0.16 L serum bottles	-	-	~200	Synthetic swine manure digestate; pH: 8	5	90	100	-	[32]
180 L HRAP interconnected to a 2.5 L AC	2.0–5.0	0.7–1.2	8–1289	Mixture synthetic wastewater/digestate (3.1:1); pH: 8.4–9.6; IC: 500–2100	23–58	85–90	-	81–94	[33]
Closed Tubular-PBR (132 L) + bubble column (2.5 L)	0.5	2.0–8.3	~715	Brunner medium (digestate mimic); pH: 9.1–9.3; IC: 1600	27	68–95	100	89–96	[34]
9.6 m^3^ HRAP interconnected to a 150 L AC	1.2–3.5	0.33–0.55	-	Simulated digestate; pH: 8.9; IC: 500	73	78–99	96–100	83–91	[21]
180 L HRAP interconnected to a 2.2 L AC	0.5	1.1	~420	Synthetic digestate; pH: 10; IC: 4458	115	95–98	98–100	95–97	[27]
180 L HRAP interconnected to a 2.5 L AC	1.0	1.0	~1417	Centrate; pH: 7–8; IC:~100	36	61–63	100	82–85	[35]
180 L HRAP interconnected to a 2.5 L AC	0.6–1.0	0.6–1.1	~1316	Centrate supplemented with IC and NPs; pH: 9.0–9.5; IC: ~1000	36	92–95	100	94–95	[36]

AC: absorption column, HRAP: high rate algal pond, HRT: hydraulic retention time, IC: inorganic carbon, MSM: mineral synthetic medium, NPs: nanoparticles, PBR: photobioreactor, RE: removal efficiency, WW: wastewater.

**Table 3:** Effect of stimulants on microalgae growth and CO_2_ removal.

Stimulant	Compound and Dose	Microalgae Strain	Carbon and Nutrient Source	Growth Rate/Productivity Increase	CO_2_ Consumption/CO_2_-RE Increase	Ref
Phytohormones	Strigolactone GR24 (10^−9^ M)	*C. vulgaris* + Fungi + Bacteria	Digestate; biogas (33.4% CO_2_)	~13.6% (growth rate), 17.8% (productivity)	~11.7% (RE)	[60]
	Strigolactone GR24 (10^−9^ M)	*Chlorella* sp. + endophytic bacteria + *Clonostachys* fungi	Digestate; biogas (35.5% CO_2_)	~52.3% (growth rate)	~24.8% (RE)	[59]
	Strigolactone GR24 (10^−9^ M)	*C. vulgaris* + AS	Digestate; biogas (33.6% CO_2_)	~32% (productivity)	~20.6% (RE)	[58]
	Cytokinin (KT) (10^−7^ M)	*Chlorella* sp. + endophytic bacteria + *Clonostachys* fungi	Digestate; biogas (35.5% CO_2_)	~46.6% (growth rate)	~13.5% (RE)	[59]
	5-Deoxystrigol (10^−11^ M)	*Chlorella* sp. + endophytic bacteria + *Clonostachys* fungi	Digestate; biogas (35.5% CO_2_)	~31.2% (growth rate)	~8.8% (RE)	[59]
	5-Deoxystrigol (10^−11^ M)	*Tetradesmus obliquus* co-culture	Digestate; biogas (33.6% CO_2_)	~53.4% (productivity)	~6.5% (RE)	[61]
Nanoparticles (NPs)	4% NPs-Fe_2_O_3_ in nanofibers (0.1 g L^−1^)	*Chlorella fusca* LEB 111	BG11; 15% CO_2_ (Indoor)	~130% (productivity)	~130% (biofixation rate)	[62]
	4% NPs-Fe_2_O_3_ in nanofibers (0.3 g L^−1^)	*Chlorella fusca* LEB 111	BG11; 15% CO_2_ (Outdoor)	~27% (productivity)	~27% (biofixation rate)	[62]
	Fe_2_O_3_ Nanorods (0.07g L^−1^)	Mixed microalgae consortium	MSM; biogas (30% CO_2_)	~38% (productivity)	~20% (cumulative consumption)	[63]
	SiO_2_ NPs (0.07g L^−1^)	Mixed microalgae consortium	MSM; biogas (30% CO_2_)	Not significant (7% productivity)	~11.5% (cumulative consumption)	[63]
	CACOI NPs (0.07g L^−1^)	Mixed microalgae consortium	MSM; biogas (30% CO_2_)	152% (productivity)	~13% (cumulative consumption)	[63]
	CALPECH CACOI NPs 8.7 wt % Fe (0.07 g L^−1^)	Mixed microalgae consortium	Digestate; biogas (29.5% CO_2_)	~109% (biomass concentration)	~7% (RE)	[64]
	CALPECH CACOI NPs (8.7 wt% Fe) (0.1 g L^−1^)	*Arthrospira platensis*	Modified Zarrouk MSM; biogas (30% CO_2_)	~61.7% (productivity)	~28% (cumulative consumption)	[65]
	SMALLOPS CACOI NPs (34.1 wt% Fe) (0.1 g L^−1^)	*Arthrospira platensis*	Modified Zarrouk MSM; biogas (30% CO_2_)	~23.3% (productivity)	~11% (cumulative consumption)	[65]
	CALPECH CACOI NPs (8.7 wt% Fe) (0.14 g L^−1^)	*Chlorella sorokiniana*	MSM; biogas (30% CO_2_)	~59% (OD final)	No significant	[66]
	CALPECH CACOI NPs in suspension (0.07–0.14 g L^−1^)	*Chlorella sorokiniana*	MSM; biogas (30% CO_2_)	Inhibitory	Negative (lower than control)	[66]
	Liquid CACOI-NPs (2 mL L^−1^)	*C. vulgaris*/*M. homosphaera* consortium	Digestate; biogas (29.5% CO_2_)	~38% (biomass concentration)	~12% (RE)	[36]

AS: activated sludge; CACOI: carbon coated zero valent iron; KT: kinetin; MSM: mineral salt medium; N.M.: Not mentioned, OD: optical density; RE: removal efficiency.

## Data Availability

No new data were created or analyzed in this study. Data sharing is not applicable to this article.

## Abbreviations

The following abbreviations are used in this manuscript:

CACOI	Carbon-Coated Zero-Valent Iron
5-DS	5-Deoxystrigol
HRAP	High Rate Algal Pond
IC	Inorganic carbon
KT	Kinetin
NPs	Nanoparticles
ppm_v_	Parts per million by volume
TRL	Technology Readiness Level
VOCs	Volatile Organic Compounds
